# Spatial and Temporal Availability of Cloud-free Optical Observations in the Tropics to Monitor Deforestation

**DOI:** 10.1038/s41597-023-02439-x

**Published:** 2023-08-22

**Authors:** Africa I. Flores-Anderson, Jeffrey Cardille, Khashayar Azad, Emil Cherrington, Yingtong Zhang, Sylvia Wilson

**Affiliations:** 1 Department of Natural Resource Sciences, McGill University, Sainte-Anne-de-Bellevue, Montreal, Quebec H9X 3V9 Canada; 2 Earth System Science Center, University of Alabama in Huntsville, Huntsville, AL USA; 3 Bieler School of Environment, McGill University, Sainte-Anne-de-Bellevue, Montreal, Quebec H9X 3V9 Canada; 4 Department of Computer Science and Software Engineering, Concordia University, Montreal, Quebec H3H 2L9 Canada; 5 Department of Earth and Environment, Boston University, Boston, MA USA; 6 National Land Imaging Program, United States Geological Service, Reston, VA USA

**Keywords:** Environmental impact, Forestry

## Abstract

State-of-the-art methodologies to monitor deforestation rely mostly on optical satellite observations. High-density optical time series can enable the detection of deforestation almost as soon as it occurs. However, deforestation monitoring in the tropics can be hindered by high cloud coverage, and thus the responsiveness of managers, enforcement agencies, and scientists. To understand the implications of cloud contamination in freely available optical data we analyzed combined time series from Landsat 7, 8, and Sentinel-2 over the tropics from 2017–2021. Datasets derived for each 30 m × 30 m of the 59.4 M km2 domain include a) number of cloud-free observations per year, b) maximum consecutive days without clear imagery within a year, and c) final date of the longest waiting period. The datasets reflect where and when data gaps in optical time series exist due to cloud contamination. Scripts to access and extend the datasets are shared and documented. The datasets can be used to prioritize areas where complementary observations, such as radar imagery, are needed for implementing effective deforestation alert systems.

## Background & Summary

The effectiveness and usability of forest monitoring systems and near-real time deforestation alerts rely on the availability of cloud-free satellite observations, particularly in the tropics where cloud coverage poses a persistent challenge to obtain useful observations. The majority of the operational forest monitoring systems used by tropical countries are based on medium spatial resolution optical satellite data, such as Landsat and more recently Sentinel-2^1–4^. Previous analyses have found that throughout the tropics, Landsat, with its 16-day repeat cycle, cannot deliver cloud-free images between 5 to 10 months in a year^5^. Others have found that regions such as the northwestern part of the Amazon are persistently covered by clouds in a 10 month period^6^. These previous analyses have not considered the increased availability of freely available optical data, not only from the multiple Landsat missions, but also from compatible observations from Sentinel-2 missions.

Identifying where and when there are observation gaps in optical time-series datasets due to cloud coverage can inform national and international programs when and where complementary datasets are needed for timely and accurate forest change detection. This is of particular relevance for near-real-time deforestation systems. For example, this information can guide where and when weather-independent observation platforms, such as Synthetic Aperture Radar (SAR) would be most useful to monitor deforestation in the tropics.

As land cover change methods evolve to combine optical and SAR data^7–9^, it is important to find critical areas where such combined methodologies are most needed, given a) the good performance of optical based systems and b) the complexity and challenges to incorporating available SAR platforms^10^. It is important to mention that currently, most of the methodologies used to monitor land cover change, including deforestation, rely mostly on optical satellite imagery^11–18^. There are several satellite sensors currently available that provide free optical imagery, which is available at a high temporal frequency. Of most importance are those of medium spatial resolution (about 20–60 m) such as the Landsat series and Sentinel-2, which are heavily used in national forest monitoring systems and near-real time deforestation alert systems^4,19^.

Therefore, in this analysis we evaluated the spatial and temporal availability of cloud-free data from the combined time series of Landsat 7, 8 and Sentinel-2, the most common sensors used to monitor deforestation over all the tropics. Table 1 lists the input datasets used to derive final datasets.

**Table 1:** Sensor data used in this analysis.

Sensors	Product level	Bands used	Reference
Landsat 7	Collection 2, Tier 1 Surface Reflectance	QA Pixel Bitmask	^21^
Landsat 8	Collection 2, Tier 1 Surface Reflectance	QA Pixel Bitmask	^29^
Sentinel-2	Surface Reflectance	Threshold 1: Image level cloud percentage	^30^
Sentinel-2	S2 Cloud Probability	Threshold 2: Cloud probability percentage	^25,26^

The main goal of the datasets created is to identify where and when there are not enough cloud-free observations which could hinder effective deforestation monitoring. The datasets developed are derived from annual time series, for the years 2017–2021, combining Landsat 7, 8 and Sentinel-2 data. The derived datasets provide information at the pixel level and include a) number of cloud-free observations, b) number of maximum consecutive days without data within a year, and c) final date in which the maximum number of consecutive days without data occurred.

These resulting datasets can provide immediate insight of areas and timing with not enough cloud-free observations and can be used in deeper analyses at country and sub-country level. To summarize the results in this paper a series of zonal statistics focused on forest extent, and deforestation fronts were performed.

## Methods

### Study area

**Fig. 1:**
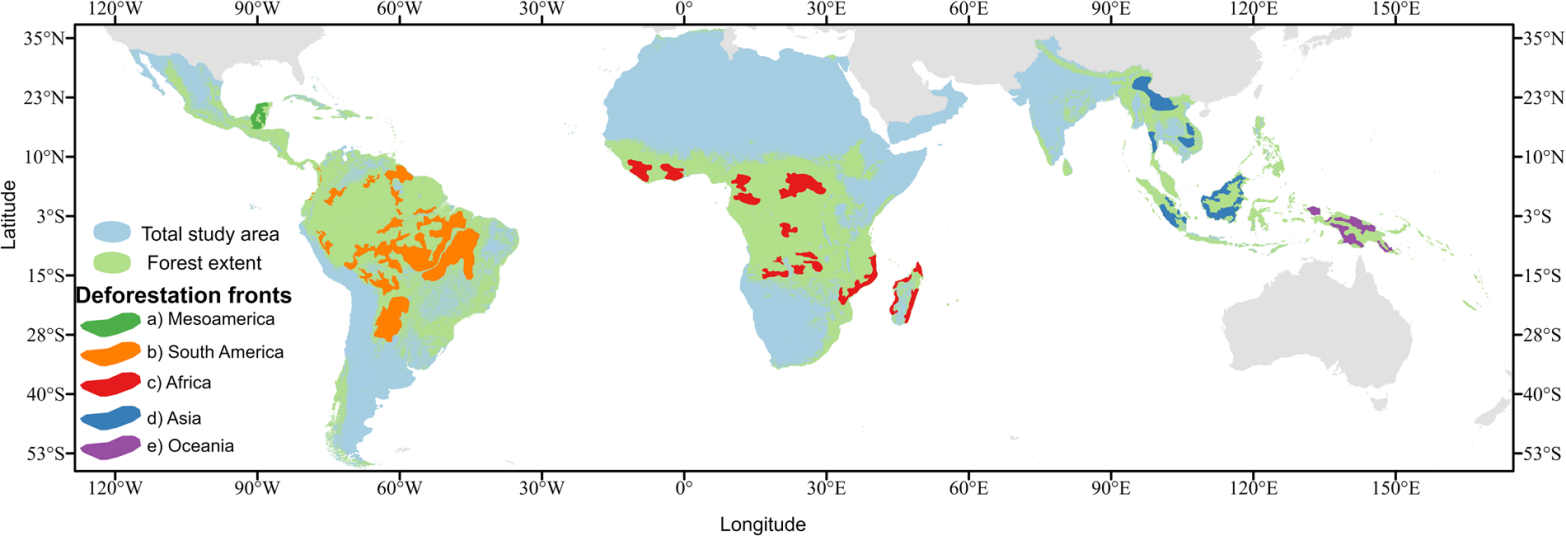
Geographic extent of this analysis and deforestation fronts. Data sources: Forest mask:^11^, Deforestation fronts^20^.

This analysis covers the global tropics using country boundaries as reference and Hansen *et al*.^11^ forest cover extent mask for year 2010. This large region contains a diverse type of forests, including temperate and tropical forests, and hosts the principal areas of deforestation fronts according to Pacheco *et al*.^20^. This analysis doesn’t include Australia, the only deforestation front per Pacheco *et al*.^20^ not included in the study, since it is outside of the pan-tropical region. See Fig. 1. The total area analyzed is of 59.4 Million Km2 and covers a total of 168 countries.

### Data and processing

Five consecutive years of satellite data from Landsat and Sentinel-2 missions were analyzed, specifically 2017–2021. All available images from Landsat 7 and 8, and Sentinel-2 for the given time period were analyzed at the pixel level over the area of interest (see Fig. 1). The main processing steps consisted of using data from the Quality Assurance (QA) band in the case of the Landsat data and cloud presence probabilities in the case of Sentinel-2, for masking out the clouds and cloud shadows at the pixel level for every year evaluated. Then the spatial and temporal availability of these cloud-free data at the pixel level were derived. The data products and bands listed in Table 1 were used to filter clouds for each respective sensor. As described in Table 1, the Surface Reflectance product level was used for the satellite data analyzed. For Landsat 7, this product is not gap-filled and contains gapped areas due to the Scan Line Corrector (SLC) failure^21^.

**Fig. 2:**
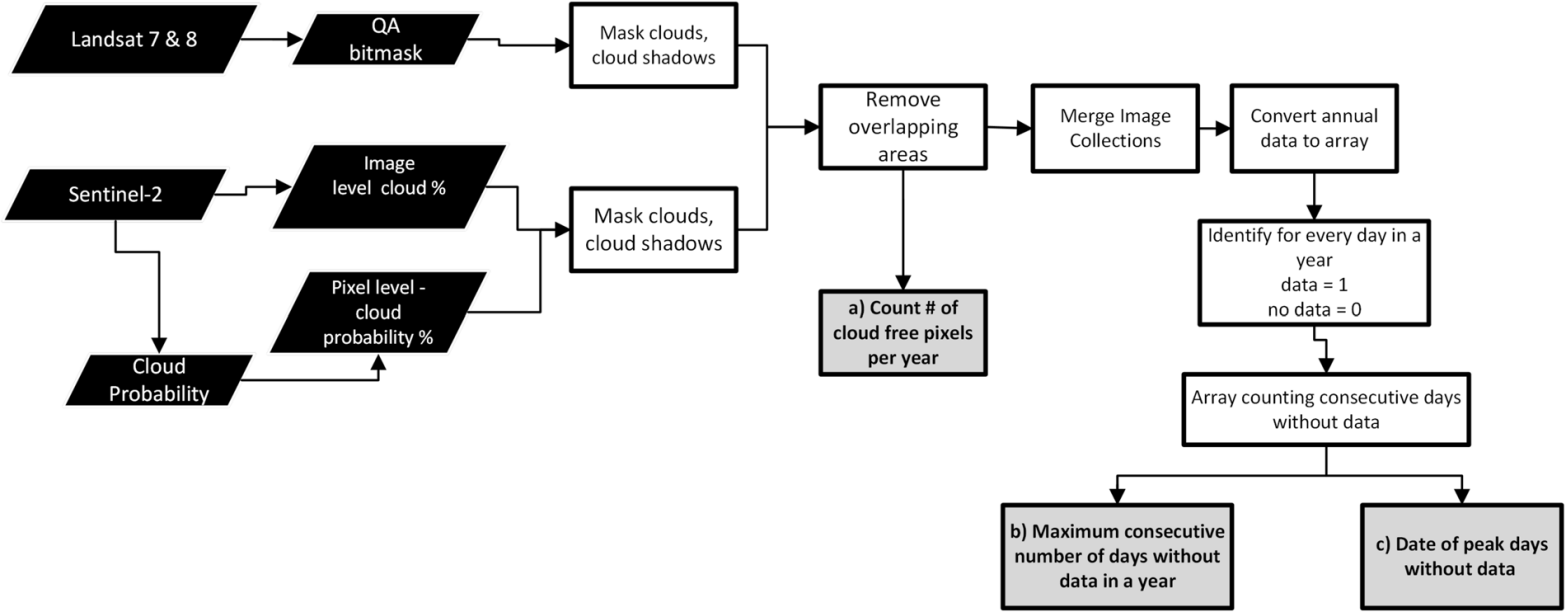
Workflow for processing satellite images to create datasets (**a**) **count**, (**b**) **maximum**, and (**c**) **date** pertinent to the spatial and temporal availability of cloud-free observations in the tropics.

All the satellite datasets used in this analysis were accessed and processed in GEE. The overall process to analyze the original datasets and create these data products is depicted in Fig. 2.

The analysis consisted of removing cloud and cloud shadows at the pixel level from the original datasets. Then, using the unique date and timing of acquisition, the overlapping areas between images along the acquisition track were discarded. For the spatial analysis these clean, -cloud-free- image collections were used to count at the pixel level the unique times, based on date information, that there was a cloud-free observation per year.

For the temporal analysis, additional processing was done to the cloud-free image collections, to merge and identify on the daily basis the presence or absence of a cloud-free observation joining all the datasets available. We interpret these combined collections in three distinct, related datasets: a) **count**, representing the number of cloud free pixels per year, b) **maximum**, representing the maximum waiting period, in days, to get a cloud-free observation, and c) **date**, representing the final date of that waiting period. Check workflow description in Fig. 2. The additional steps for the spatial and temporal analysis are described in detail in the sections below.

Further analysis was done at the regional deforestation front level to derive information about where and when there is not availability of cloud-free data to depict data usability.

#### Spatial availability of cloud-free pixels

In GEE, the number of unique observations were calculated from the cloud-free image collections for all the sensors. This produced the number of cloud-free observations per year for all combined sensors, as listed in product a) **count** in Fig. 2. The final datasets were produced at a nominal resolution of 30 m, for all the study region. Additional post-processing included statistical analysis of the data created.

#### Temporal availability of cloud-free pixels

Based on the cloud and cloud-shadow free image collections, and using the date information a new dataset was created that combined all sensor data, and identify data availability on the daily basis for over a year. Finally, the annual arrays are processed to extract two variables, a) maximum number of consecutive days without cloud-free data and b) final date of that maximum waiting period. These two variables were selected to understand the temporal availability of cloud-free data over the tropics. See Fig. 2. The temporal datasets created provide unique and novel temporal information that has not been created before at such geographic scale, spatial resolution (30 m), and combining multiple med-resolution satellite data sources. This is the first time that such temporal information on cloud-free data availability is being produced. Previous studies have focused on spatial data distribution and availability and rely on a solely data source^22^.

## Data Records

The datasets created respond to where and when there are gaps in optical satellite observations due to cloud contamination at 30 m × 30 m pixel level in a year, and are based on a spatial and temporal distribution analysis. We are creating three datasets 1) **count**, representing the number of cloud free pixels per year, 2) **maximum**, representing the maximum waiting period, in days, to get a cloud-free observation, and 3) **date**, representing the final date of that waiting period. Dataset 1) is derived from the spatial distribution analysis and 2) and 3) from the temporal distribution analysis.

The final data outputs of this analysis were created at an annual scale for 5 years, 2017 to 2021, and are available as GEE assets as listed in Table 2. The spatial distribution of cloud-free data contains one band, ‘valid_obs’ which indicates the number of cloud-free observations at the pixel level for a given year. The temporal data contains two bands, ‘max’ and ‘Max_Day’, the first band indicates in days the maximum waiting period to get a cloud-free observation per year, and the second band indicates the date at the end of that waiting period.

**Table 2:** Spatial and temporal cloud-free data availability.

Dataset	Access location	Band name	Data type	Range	Code to visualize
Spatial Distribution	ee.Image(‘projects/sentinelati-1547136065067/assets/ValidObs/all_ValidObs2017’ ee.Image(‘projects/sentinelati-1547136065067/assets/ValidObs/all_ValidObs2018’ projects/sentinelati-1547136065067/assets/Continent/	valid_obs	Int64	0, 150	^24^
Temporal Distribution	projects/sentinelati-1547136065067/assets/Temporal03 users/africa_uah/Temporal03	max MaxDay	64Bit	0-700 1-365	^24^

Due to the large size of the data (~1 TB), it is available via GEE, however a subset is available at Zenodo^23^, accessible at (10.5281/zenodo.7714192^23^.

The spatial datasets for the years 2017 and 2018, are available for the whole study region as Earth Engine images. For the years 2019–2021, the spatial data is available in regional subsections. The temporal datasets were created as ee.ImageCollections per year. The asset repository to access these datasets is listed in Table 2. Scripts to access and read the spatial and temporal cloud-free distribution data are available in GitHub at (10.5281/zenodo.7761963)^24^.

## Technical Validation

The quality of the results is heavily dependent on the accuracy of the cloud and cloud shadow detection algorithms used in the input datasets. In the case of the Landsat datasets, the cloud information used in this study is generated using the CFMask v3.3.1^21^. In the case of the Sentinel-2 data the S2cloudless^25^ algorithm is used, check also Table 1. Previous studies have documented the accuracy of these cloud masking algorithms, with Skakun *et al*.^26^ reporting for S2cloudless overall accuracies between 85.2% to 93.1% for Sentinel 2 data, depending on reference dataset used. Qiu *et al*.^27^ reports for FMask v3 an overall accuracy of 90.73% and commissions errors in mountainous regions are noted as known issues.

An independent technical assessment was performed on the spatial dataset, **count**, which contains the information on number of valid observations (valid_obs), since the error accounted here will expand to the temporal datasets b) **maximum** and c) **date**. The steps for the technical analysis include:

1. Check data distribution of the **count** dataset and select representative values for low, typical, and high cloud-free data availability
2. Randomly sample 100 observations per representative value
3. Count the truth cloud-free observations per sensor for the random samples
4. Calculate final statistics comparing the truth counting with the calculated value for number of cloud-free observations

### Step 1

A year that represents the current state of data availability was selected, in this case 2021. Then, based on the data distribution of the **count** dataset for 2021, three representative values were selected, the first quartile (value 32) representing low data availability; the median (value 49) representing typical availability; and the third quartile (value 63), representing high availability. Table 3 summarizes the data distribution for each of the years evaluated, and it is evident that these values represent the distribution of the data for the last 3 years evaluated (2019–2021).

**Table 3:** Summary distribution of the dataset **count** for all the years.

Year	Min.	1st Qu.	Median	Mean	3rd Qu.	Max.
2017	0	11	15	15.71	19	94
2018	0	12	17	17.67	21	162
2019	0	33	49	50.22	63	182
2020	0	34	50	50.91	63	192
2021	0	32	49	49.61	63	184

### Step 2

**Fig. 3:**
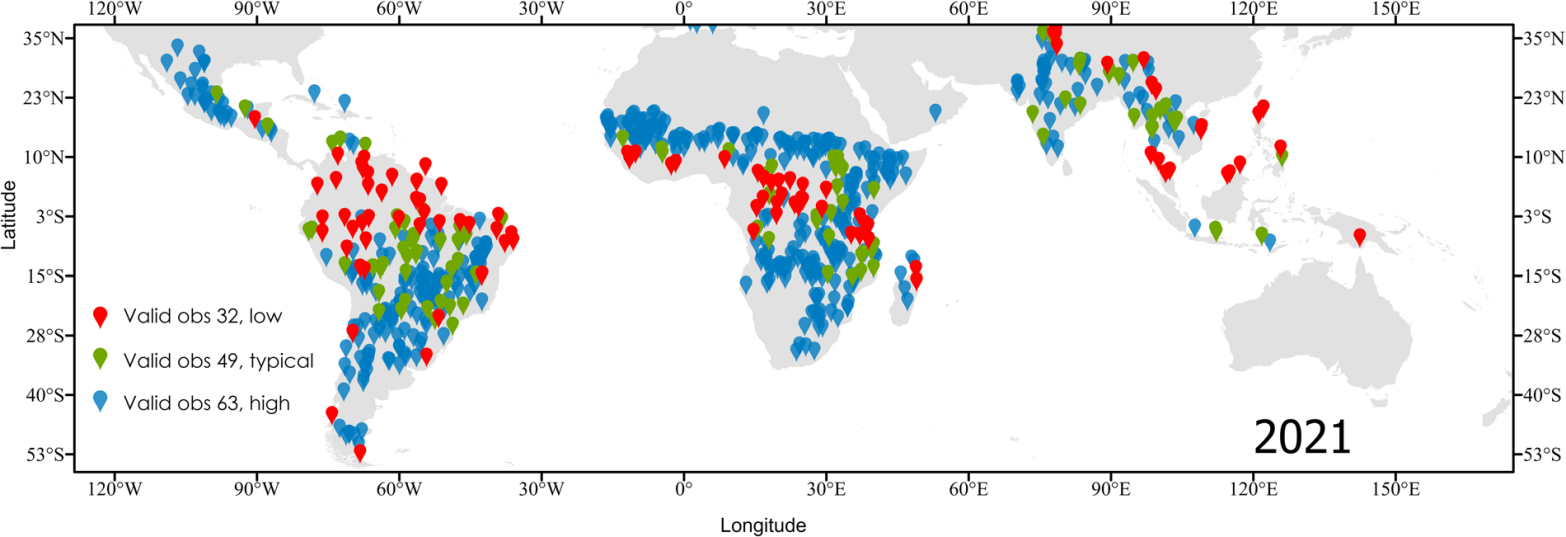
Location of the samples assessed in the technical validation. Each color represents a key value of the data distribution of the dataset (**a**) **count**. Red dots represent low data availability, green represent locations with typical data availability, and blue dots represent locations with high data availability.

Followed, 100 points were selected randomly for each of these 3 key values. Figure 3 displays the random samples selected.

### Step 3

Then, using all the images available from Landsat 7, 8 and Sentinel-2 for the year 2021, the number of cloud and shadow free observations were counted visually for each of the 300 total locations selected randomly in GEE. Our assessment to count a valid observation followed this criteria:

- All clear observations where the Earth’s surface is clearly visible were counted as valid observations
- Very thin clouds and semi-transparent clouds that allow visibility of the Earth’s surface, where deforestation detection is still feasible, were considered valid observations
- Thick, and thin clouds that obstructed visibility of the Earth’s surface were considered clouds, i.e. not valid observations
- All shadows were not counted as valid observation

### Step 4

The following statistics were calculated to assess the quality of the results, for each sensor and all combined sensors: a) Mean Absolute Error (MAE), b) Bias, and c) Relative Bias or Percent Bias. The following equations were used to calculate these metrics:

1 $$MAE=\frac{1}{n}\mathop{\sum }\limits_{i=1}^{n}\left|{y}_{i}^{count}-{y}_{i}^{calc}\right|$$

2 $$BIAS=\frac{1}{n}\mathop{\sum }\limits_{i=1}^{n}\left({y}_{i}^{count}-{y}_{i}^{calc}\right)$$

3 $$PBIAS=\frac{\mathop{\sum }\limits_{i=1}^{n}({y}_{i}^{count}-{y}_{i}^{calc})}{\mathop{\sum }\limits_{i=1}^{n}({y}_{i}^{count})}$$

Where ${y}_{i}^{count}$ is the *i*_*th*_ visually counted value for cloud-free observations, ${y}_{i}^{calc}$ is the *i*_*th*_ calculated value for number of cloud-free observations by our **count** dataset.

The final statistics are listed in Table 4. For Landsat with a MAE of 4.37, 6.35, and 6.29 for each of the quartiles, respectively, indicates a typical error of 5 observations, and a relative bias of 23.42% that is, it typically excludes 22%, 26%, and 21% of the pixels, for relatively cloudy, typical tropical, and relatively clear tropical areas, respectively. This means that the standard cloud masking used in the Landsat data is excluding overall about 23.42% of the pixels that are actually useful for mapping deforestation, per counting criteria described in Step 3. In contrast, the assessment of Sentinel-2 indicates that the number of cloud-free observations derived from it is slightly overestimated. With an overall MAE of 2.92 number of observations, that is, it typically adds about 6.24% of observations. This error is more significant in cloudier areas, with a relative bias of 13.79%. The overall accuracy of our results indicate a low error, of about 5.47 observations for the **Count** dataset, which integrates, both Landsat and Sentinel-2 data, this error derives mostly from the Landsat dataset. Across the whole dataset **Count**, we have a bias of 3.21 observations, indicating that about 3 observations or 6% are excluded or not considered.

**Table 4:** Summary statistics to assess the accuracy of the valid observations, **count**, dataset.

Sensor	MAE	Bias	Rel Bias %
	Low data availability		
Landsat	4.37	3.71	22.11
Sentinel-2	2.39	−2.29	−13.79
All	3.7	1.42	4.25
	**Typical data availability**		
Landsat	6.35	5.76	26.65
Sentinel-2	2.93	−0.70	−2.15
All	6.85	5.07	9.39
	**High data availability**		
Landsat	6.29	5.69	21.56
Sentinel-2	3.43	−2.57	−6.48
All	5.81	3.12	4.72
	**For all dataset**		
Landsat	5.68	5.06	23.42
Sentinel-2	2.92	−1.85	−6.24
All	5.47	3.21	6.28

**Fig. 4:**
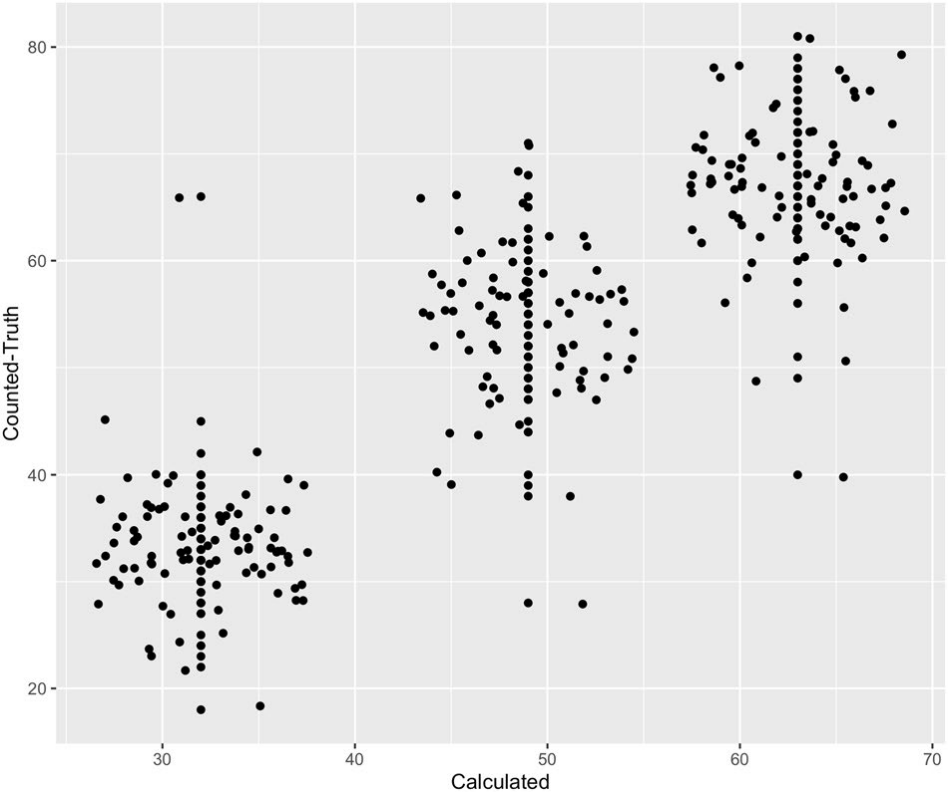
Valid Observation Assessment showing in the x-axis the number of cloud-free pixels as calculated by our dataset **count** and in the y-axis the counted cloud-free observations (truth) collected following the technical validation steps.

Figure 4 displays the distribution of the counted “truth” cloud-free observations (*y* axis) compared to the expected value calculated in the dataset **Count**, (*x* axis). This graph portrays how overall, our calculated dataset for **Count** slightly underestimates the number of cloud-free observations, per criteria used to count cloud and shadow free pixels explained in Step 3. The big outlier in the calculated value 32, which actually had 66 cloud-free observations, represents a mountainous region seasonally covered by snow, which reinforces the finding of Qiu *et al*.^27^ that FMask v3 is too constraining particularly in mountainous areas. This technical assessment also indicates that most of the error is coming from the Landsat dataset, see Table 4.

### Implications

The datasets created provide a practical view of the number of cloud-free observation and the wait period in rapidly changing areas. Users who want a general view of the landscape can get it most of the time in most places using the spatial and temporal datasets created.

The first two years evaluated, 2017 and 2018, are dominated by Landsat data, and beginning in 2019 there is more consistent presence of Sentinel-2 data. Hence, for those two initial years 2017 and 2018, the MAE and Bias derived from Landsat is more representative. The MAE and Bias that should be used to assess the quality of the data is the one listed in the raw ‘All’ since, the datasets created are derived from the combined image collections of Landsat and Sentinel-2. The lowest error and bias are found in relatively cloudy areas, with a bias of 1.47 observations, which indicates that the temporal datasets **Maximum** and **Date** are also more accurate in those regions. The new version of FMask, v4^27^ may rectify the known issues in the cloud masking of Landsat data in Collection 2. Please note that for the creation of these datasets we are using the most current available data from Landsat and Sentinel-2.

## Usage Notes

Use of the final data at the country and sub-country level is encouraged, this will provide useful insight when developing and assessing near-real time monitoring systems that rely on optical data in this new era of high data availability. The intended use of the spatial cloud-free distribution data is first to inform how many optical cloud-free observations are available in a given year at the pixel level. This can further inform the quality of derived products using these optical data. Zhang *et al*.^28^ identified that at least 7 clear observations per year could provide a good time-series analysis result. Hence, the spatial dataset of **count** can be used to further discern areas with enough cloud-free observations for proper change detection using time-series.

In addition, part of the methodology to create the cloud-free image collections can be used as input data for any future analysis.

The temporal datasets can be used to understand data needs and prioritize additional tasking and/or use of complementary datasets, from either commercial or public sources. Particularly, understanding when those complementary datasets would be needed in a given region. From the deforestation perspective, these datasets provide a good insight of where and when near-real time deforestation systems could be under-performing and can benefit the most from additional satellite datasets. For example, the average waiting time to get cloud-free data for the Oceania deforestation front is about 120 days, and for the Asia deforestation front is about 100 days. In the Africa deforestation front, the critical months to complement optical observations with SAR or other dataset is between August and November. In the South America deforestation front, cloud-free data are more likely during October and November, leaving fewer cloud-free observations between March and May. Such insight can be derived from our temporal datasets **maximum** and **date** at any geographic scale, be it local, national or regional. A public web interface for rapid exploration and visualization of the data at the country level is available at https://africa-uah.users.earthengine.app/view/spatialtemporalcloudfree).

We also provide code examples for accessing and processing the final results in Earth Engine Code Editor, a) for the spatial cloud-free distribution results: (https://code.earthengine.google.com/de193db53d27cf1c2ab061e40de8f6bd and b) for the temporal results: https://code.earthengine.google.com/fda2bb4b06f08b6020a541dafe9e2e3d.

## Data Availability

To ensure reproducibility, and recreate the results for other regions and/or years the example code for creating and accessing all the spatial and temporal datasets is available in Zenodo at (10.5281/zenodo.7761963)^24^.

## References

[1] Finer B (2018). Combating deforestation: From satellite to intervention. Science.

[2] Tarazona Y, Mantas V, Pereira A (2018). Improving tropical deforestation detection through using photosynthetic vegetation time series – (pvts-. Ecol. Indic.

[3] Kalamandeen M (2018). Pervasive Rise of Small-scale Deforestation in Amazonia. Scientific Reports.

[4] Vargas C, Montalban J, Leon A (2019). Early warning tropical forest loss alerts in peru using landsat. Environ. Res. Commun.

[5] Hoekman D, Quinones M, Vissers M (2010). K& c science report – phase 1 tropical forest and wetlands mapping, case study borneo. ALOS Kyoto Carbon Initiat. Sci. Team Reports Phase.

[6] Martins V (2017). Seasonal and interannual assessment of cloud cover and atmospheric constituents across the amazon (2000–2015): Insights for remote sensing and climate analysis. *ISPRS J. Photogramm*. Remote Sens.

[7] Hirschmugl, M. *et al*. Use of sar and optical time series for tropical forest disturbance mapping. *Remote Sens* **12**, 10.3390/rs12040727. (2020).

[8] Reiche J, de Bruin S, Hoekman D, Verbesselt J, Herold M (2015). A bayesian approach to combine landsat and alos palsar time series for near real-time deforestation detection. Remote Sensing.

[9] Abramowitz, J., Cherrington, E., Griffin, R., Muench, R. & Mensah, F. Differentiating Oil Palm Plantations from Natural Forest to Improve Land Cover Mapping in Ghana. *Remote Sensing Applications: Society and Environment* 100968, 10.1016/j.rsase.2023.100968 (2023).

[10] Hethcoat, M. G., Carreiras, M. B., Bryant, R. G., Quegan, S. & Edwards, D. P. Combining Sentinel-1 and Landsat 8 Does Not Improve Classification Accuracy of Tropical Selective Logging. 1–15 (2022).

[11] Hansen MC (2013). High-resolution global maps of 21st-century forest cover change. Science.

[12] Cohen, W. B. *et al*. How Similar Are Forest Disturbance Maps Derived from Different Landsat Time Series Algorithms? *Forests* **8**, 10.3390/f8040098 (2017).

[13] Zhu, Z. *et al*. Continuous monitoring of land disturbance based on Landsat time series. *Remote Sensing of Environment* **238**, 10.1016/j.rse.2019.03.009 (2020).

[14] Zhu Z, Woodcock C (2014). Continuous change detection and classification of land cover using all available landsat data. Remote Sens. Environ.

[15] Chen, S. *et al*. Monitoring temperate forest degradation on Google Earth Engine using Landsat time series analysis. *Remote Sensing of Environment* **265**, 10.1016/j.rse.2021.112648 (2021).

[16] Cohen WB, Yang Z, Healey SP, Kennedy RE, Gorelick N (2018). A LandTrendr multispectral ensemble for forest disturbance detection. Remote Sensing of Environment.

[17] Saah D (2020). Primitives as building blocks for constructing land cover maps. International Journal of Applied Earth Observation and Geoinformation.

[18] Cardille J, Fortin J (2016). Bayesian updating of land-cover estimates in a data-rich environment. Remote Sens. Environ.

[19] Finer, M., Villa, L. & Mamani, N. Real-time amazon fire monitoring app. maap. https://maaproject.org/2020/amazon-fire-app/ (2020).

[20] Pacheco, P. Deforestation fronts: Drivers and responses in a changing world. Available at https://files.worldwildlife.org/wwfcmsprod/files/Publication/file/ocuoxmdil_Deforestation_fronts___drivers_and_responses_in_a_changing_world___full_report__1_.pdf (2021).

[21] U.S. Geological Survey USGS. Landsat 4-7 Level 2 Science Product (L2SP) Guide September 2021. Tech. Rep. September (2021).

[22] Zhang, Y. *et al*. A Global Analysis of the Spatial and Temporal Variability of Usable Landsat Observations at the Pixel Scale. *Frontiers in Remote Sensing* **3**, 10.3389/frsen.2022.894618 (2022).

[23] Flores-Anderson AI (2023). Zenodo.

[24] Flores-Anderson A (2023). Zenodo.

[25] Batič, M. Sentinel Hub Cloud Detector — s2cloudless. *Medium* https://medium.com/253 sentinel-hub/sentinel-hub-cloud-detector-s2cloudless-a67d263d3025 (2018).

[26] Skakun, S. *et al*. Remote Sensing of Environment Cloud Mask Intercomparison eXercise (CMIX): An evaluation of cloud masking algorithms for Landsat 8 and Sentinel-2. **274**, 10.1016/j.rse.2022.112990 (2022).

[27] Qiu S, Zhu Z, He B (2019). Fmask 4.0: Improved cloud and cloud shadow detection in Landsats 4–8 and Sentinel-2 imagery. Remote Sensing of Environment.

[28] Zhang J, Shang R, Rittenhouse C, Witharana C, Zhu Z (2021). Evaluating the impacts of models, data density and irregularity on reconstructing and forecasting dense landsat time series. Science of Remote Sensing.

[29] U.S. Geological Survey USGS. Landsat 9 Data Users Handbook Landsat 9 Data Users Handbook. Tech. Rep. February (2022).

[30] (ESA), E. S. A. & Space, T. A. Sentinel-2 products specification document. Available at https://sentinel.esa.int/documents/247904/685211/sentinel-2-products-specification-document (2021).

